# Clinical Benefits of Invasive Strategy in Stable Angina Patients with Low Systolic Blood Pressure: A Post Hoc Analysis of the ISCHEMIA Trial

**DOI:** 10.3390/jcm15062100

**Published:** 2026-03-10

**Authors:** Yicong Ye, Li Lin, Mengge Zhou, Yaodong Ding, Yang Zhang, Zehao Zhao, Wenjie Wang, Xiliang Zhao, Yong Zeng

**Affiliations:** 1 Beijing Anzhen Hospital, Capital Medical University, Beijing 100029, China; yicongye@mail.ccmu.edu.cn (Y.Y.); linli1061800792@163.com (L.L.); dingyaodong1@163.com (Y.D.); waterfallyang@163.com (Y.Z.); cardiozhao@163.com (Z.Z.); 13120043661@163.com (W.W.); 2 Department of Epidemiology and Statistics, Institute of Basic Medical Sciences Chinese Academy of Medical Sciences, School of Basic Medicine Peking Union Medical College, Beijing 100005, China; mengge@ibms.pumc.edu.cn

**Keywords:** stable angina, systolic blood pressure, invasive strategy, percutaneous coronary intervention, revascularization, optimal medical therapy

## Abstract

**Background:** The ISCHEMIA trial demonstrated no overall prognostic benefit of an initial invasive strategy over optimal medical therapy (OMT) in patients with chronic coronary syndrome (CCS) and moderate-to-severe ischemia. However, managing patients with stable angina and low systolic blood pressure (SBP) remains challenging due to limited tolerance to vasodilatory anti-anginal drugs and the uncertain role of revascularization in improving long-term outcomes for this subgroup. **Objectives:** This study aimed to estimate the treatment effect of an initial invasive strategy (INV) compared with conservative medical therapy (CON) on long-term clinical outcomes and quality of life in patients with stable angina, particularly those with low baseline systolic blood pressure (≤120 mmHg). **Methods:** We conducted a post hoc analysis of 3544 patients with stable angina from the ISCHEMIA trial, divided into an initial invasive strategy or a conservative approach. The primary endpoint was a 3-year composite of cardiovascular death, myocardial infarction, hospitalization for unstable angina or heart failure, or resuscitated cardiac arrest. Health-related quality of life was assessed using the Seattle Angina Questionnaire (SAQ). In the subgroup, patients were stratified by baseline SBP, diastolic blood pressure (DBP) and heart rate; the Cox model was adjusted for the covariates. **Results:** Baseline characteristics were generally comparable between the two groups. Over 3 years of follow-up, no significant difference in primary endpoint events was observed between the INV and CON group in the overall cohort (HR = 0.94, 95%CI 0.77–1.14, *p* = 0.53), and the INV group had the higher SAQ score. Among patients with low baseline SBP (≤120 mmHg), after adjusting for clinical factors using Cox regression, randomized treatment assignment to the INV approach significantly reduced adverse cardiovascular events compared with conservative therapy (HR = 0.58, 95%CI 0.38 to 0.89). **Conclusions:** In patients with stable angina, an invasive strategy improved long-term quality of life. Among those with low baseline SBP (≤120 mmHg) and limited tolerance to vasodilatory anti-anginal drugs, invasive management reduced 3-year adverse events, supporting tailored revascularization strategies for these patients; a larger cohort is needed for validation. However, this subgroup-specific causal contrast derives from a post hoc exploratory analysis and should be interpreted cautiously; prospective randomized studies are needed to further validate these findings.

## 1. Introduction

The main findings of the ISCHEMIA trial demonstrated that, compared with optimal medical therapy (OMT) alone, the addition of an initial invasive strategy did not confer additional benefit in patients with chronic coronary syndrome (CCS) and moderate-to-severe myocardial ischemia [1,2,3]. OMT remains the cornerstone of management for patients with CCS; one secondary analysis from the ORBITA-2 showed that in CCS patients with typical angina symptoms, percutaneous coronary intervention (PCI) could significantly improve symptom burden and quality of life [4]. However, the additional benefits of revascularization on symptom relief and long-term prognosis, when added to OMT, remain a subject of ongoing debate [5,6,7].

Two prospective trials have demonstrated the cardiovascular benefits of intensive blood pressure lowering [8,9], and retrospective studies have suggested that normotension prior to PCI is associated with a reduced risk of in-stent restenosis (ISR) [10]. Nevertheless, the potential adverse effects of excessively low blood pressure cannot be overlooked, particularly among patients at high cardiovascular risk. An overly reduced blood pressure may compromise the body’s autoregulatory capacity to maintain adequate perfusion of vital organs [11]. Meanwhile, guideline-recommended first-line anti-anginal medications, such as β-blockers and calcium channel blockers (CCBs) [12,13], tend to lower blood pressure, creating a therapeutic dilemma: in patients with coronary artery disease (CAD) and low blood pressure, identifying an appropriate balance between angina control and hemodynamic stability remains a clinical challenge. Current guidelines provided limited evidence or specific recommendations on this issue [14,15,16].

The purpose of this study is to conduct a secondary analysis using the ISCHEMIA study to identify patterns in the use of anti-angina drugs, preoperative blood pressure levels (especially systolic blood pressure at baseline), and revascularization outcomes, providing a revascularization approach for patients who are intolerant to anti-anginal medications.

## 2. Methods

### 2.1. Overall

This post hoc analysis was conducted in accordance with the CONSORT 2010 statement guidelines and involved a pooled dataset derived from the ISCHEMIA study (NCT01471522) [1,17] (Supplementary Materials). The data utilized in this study were sourced from the Biologic Specimen and Data Repository Information Coordinating Center of the National Heart, Lung, and Blood Institute (NHLBI) [18]. In summary, the ISCHEMIA trial enrolled individuals diagnosed with chronic coronary syndrome who exhibited moderate-to-severe ischemia on clinically indicated stress imaging, or severe ischemia identified during exercise testing. After initial noninvasive ischemia testing, all patients underwent coronary computed tomography angiography (CCTA) to exclude significant left main coronary artery disease and to confirm the presence of coronary artery disease, in accordance with the ISCHEMIA trial protocol. Eligibility criteria required participants to be clinically stable, whether presenting with stable angina or asymptomatic (silent) ischemia. The variables used in this analysis included baseline demographic characteristics, clinical characteristics, adjudicated clinical endpoints as defined by the ISCHEMIA trial protocol, and health-related quality of life assessed using the Seattle Angina Questionnaire (SAQ) at baseline and at 3-year follow-up. The key exclusion criteria included an estimated glomerular filtration rate (eGFR) below 30 mL/min/1.73 m^2^, a recent acute coronary syndrome, unprotected left main coronary artery stenosis of ≥50%, left ventricular ejection fraction (LVEF) less than 35%, New York Heart Association (NYHA) class III or IV heart failure, and refractory angina despite optimal medical therapy at the maximum tolerated doses [19]. The study adhered to the principles of the Declaration of Helsinki and obtained approval from the Institutional Review Boards.

### 2.2. Study Population

All patients with angina pectoris from the ISCHEMIA trial were included and categorized into the invasive strategy group and the conservative strategy group. In addition, we further stratified these patients based on their baseline systolic blood pressure (the cut-off value refers to previous research content, as follows (not a prespecified study endpoint): SBP < 120 mmHg vs. SBP ≥ 120 mmHg [20]) to explore the incidence of clinical outcomes across different blood pressure levels. For patients with low pressure (<120 mmHg), a further subgroup analysis was performed by treatment strategy (invasive vs. conservative) to identify factors potentially driving the occurrence of adverse clinical events. Baseline and follow-up data were collected for all patients, including age, sex, race, vital signs, medication use, imaging findings, angina characteristics, laboratory test results, and medical history. The cut-off values for low diastolic blood pressure (DBP) and low heart rate used in the subgroup analyses were determined based on guideline recommendations [14].

### 2.3. Outcomes

The study endpoints were defined in accordance with those of the ISCHEMIA trial. The primary clinical endpoint of this study was the composite of cardiovascular death, myocardial infarction, hospitalization for unstable angina, heart failure, and resuscitation after cardiac arrest at the 3-year follow-up. The secondary clinical endpoints comprised each individual component of the primary composite outcome, including cardiovascular death, myocardial infarction, unstable angina, heart failure, and resuscitation after cardiac arrest. The health-related quality of life was the SAQ assessed after randomization. In an exploratory analysis, we examined the interaction between SBP, DBP, and heart rate with the primary clinical endpoint, respectively.

### 2.4. Statistical Analyses

Baseline characteristics were presented as counts and percentages (%) for categorical variables, and as medians with interquartile ranges (IQRs) for continuous variables. Group comparisons at baseline were performed using Fisher’s exact test for categorical data and the Kruskal–Wallis test for continuous data, as appropriate. Time-to-event outcomes were assessed using Kaplan–Meier survival analysis. Cox proportional hazards regression analysis was used to calculate the hazard ratio (HR) and 95% confidence interval (CI) to compare the risk of clinical events between subgroups. To assess the proportional hazards assumption, Schoenfeld residuals from the fitted models have been calculated. To account for differences between the groups, the primary analysis was based on a Cox model adjusted for the covariates that represent the most important risk factors for the development of CAD, including age, sex, degree of ischemia, smoking status, hypertension, diabetes, prior MI, PCI, or CABG, history of heart failure, and continuous ejection fraction [21,22]. All adjusted analyses were conducted within an intention-to-treat framework. The primary causal estimand of interest was the intention-to-treat effect, defined as the effect of assignment to an initial invasive strategy versus a conservative strategy based on the original randomization. All statistical analyses were carried out using R version 3.6.1 (The R Foundation, Vienna, Austria). A two-sided *p* < 0.05 was statistically significant.

## 3. Result

### 3.1. Study Participants

**Figure 1:**
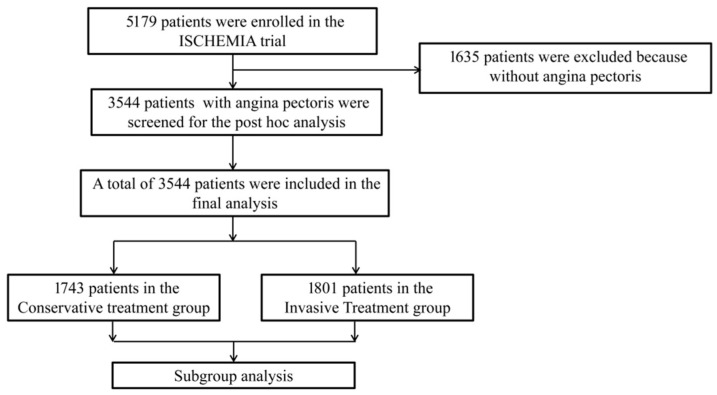
Study flowchart. All analyses shown represent post hoc secondary analyses of the ISCHEMIA randomized clinical trial.

Figure 1 provides the flowchart of this study. There were 1743 patients in the conservative treatment group (CON) and 1801 patients in the invasive treatment group (INV). The baseline characteristics of all participants are listed in Table 1. The overall median age was 62.62 years (SD 9.54), without clinically relevant group differences. There were no significant differences in the distribution proportions of race, gender, smoking history, clinical history, vital signs and laboratory tests between the two groups of patients. The degree of ischemia and coronary artery stenosis between the CON and INV groups was comparable, but patients in the CON group reported a more favorable angina status, particularly reflected by a higher SAQ-7 Quality of Life Score compared with the INV group (Table 1).

### 3.2. Clinical Outcome

**Figure 2:**
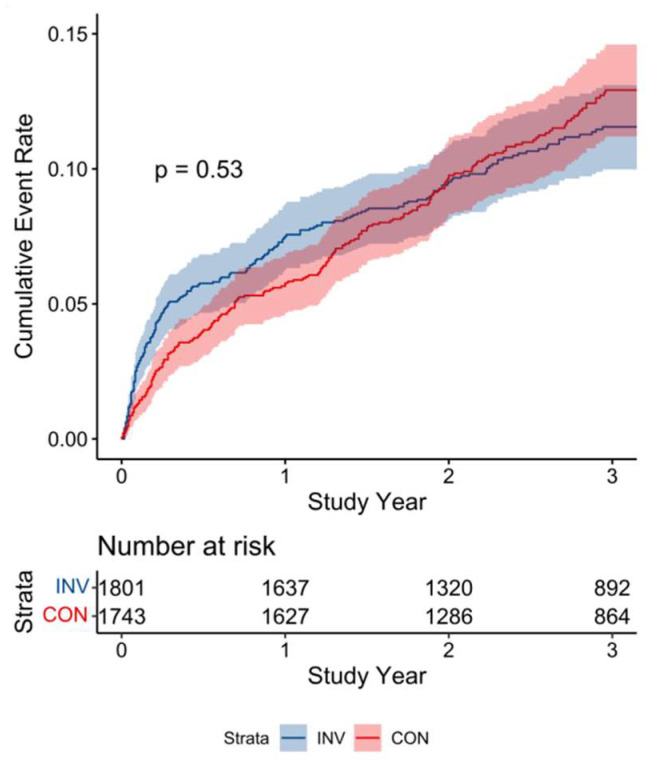
The time-to-event curves of the primary outcome at 3-year follow-up. All analyses shown represent post hoc secondary analyses of the ISCHEMIA randomized clinical trial.

The primary outcomes occurred in 192 (10.7%) of 1801 patients in the INV group and 201 (11.5%) of 1743 patients in the CON group (HR:0.94, 0.77–1.14 95%CI, *p* = 0.53) at 3-year follow-up. The cumulative event rates and estimated differences at 6-month, 1-year, 2-year, and 3-year follow-up are shown in Table 2 and Figure 2. There was no difference in the secondary endpoint between the two groups for cardiovascular death, myocardial infarction, hospitalization for unstable angina, heart failure and resuscitation after cardiac arrest at the 3-year follow-up (Table 3). However, we found that the incidence of hospitalization for heart failure in the INV group was higher than that in the CON group at 3-year follow-up (1.17% vs. 0.46%, *p* = 0.0235). Health-related quality-of-life outcomes were mainly assessed by the SAQ; even the baseline angina status was broadly comparable, while patients in the INV group experienced a lower frequency of angina episodes and reported better quality of life at the 3-year follow-up (Table 4).

### 3.3. Exploratory Analysis

**Figure 3:**
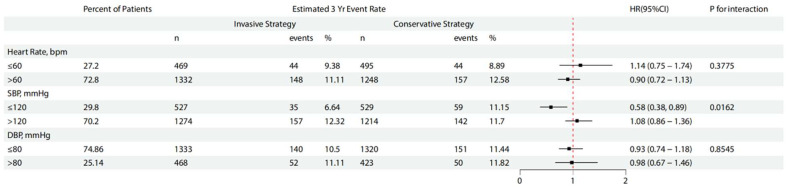
Subgroup analyses for the primary endpoint. SBP, systolic blood pressure; DBP, diastolic blood pressure. All analyses shown represent post hoc secondary analyses of the ISCHEMIA randomized clinical trial.

Table 5 and Figure 3 show the estimated risks associated with the primary outcomes across multiple subgroups. We observed that among patients with baseline SBP levels ≤ 120 mmHg (low SBP), an invasive treatment strategy was associated with a markedly lower incidence of 3-year event rate compared with a conservative strategy (HR = 0.58, 95%CI 0.38 to 0.89). Conversely, for patients with baseline SBP levels > 120 mmHg, the invasive strategy did not confer a significant advantage over the conservative approach (*p* for interaction = 0.0162).

Table 6 further explores the proportion of each component in the primary endpoint of the low SBP group patients. We found that the highest proportion of primary endpoints was hospitalization for MI, especially in the CON group (3.98% vs. 7.37%, *p* = 0.0255). Table 7 shows that the low SBP (≤120) group had the lowest proportion of patients taking ACEI/ARB, β-blockers, and CCBs, with similar findings observed both at baseline and at the last visit. However, these subgroup findings should be interpreted cautiously and do not alter the neutral overall treatment effect.

Table 8 presents the medication usage during baseline and follow-up periods in the low SBP group, in which the age, gender, and use of ACEI/ARB, β-blocker, and CCB during the baseline period were generally similar, but the overall proportion of anti-anginal medications in the INV group was lower than that in the CON group (46.5% vs. 54.3%, *p* = 0.041). Based on the results from the last visit, the INV group had a lower proportion of patients taking anti-anginal medications (12.3% vs. 24.8%, *p* < 0.001), and the proportion of taking long-acting nitrates showed a further reduction compared with the CON group (9.5% vs. 18.9%, *p* < 0.001).

## 4. Discussion

In this study, we identified the following key findings: 1. For the ischemic patients with angina pectoris, there was no significant difference in the incidence of primary endpoint events at the 3-year follow-up between those receiving invasive versus conservative treatment strategies. However, patients in the invasive treatment group experienced a lower prevalence of angina and reported better quality of life at the 3-year follow-up; 2. Subgroup analysis revealed that, in the low SBP group (≤120 mmHg), patients who received invasive treatment had a lower proportion of anti-anginal medication use and a reduced incidence of primary endpoint events. It suggests that, for patients with stable angina who cannot tolerate anti-anginal medications due to low blood pressure, an invasive treatment strategy may provide greater clinical benefits compared with a conservative treatment approach. The novelty of this study lies in its focused hypothesis-driven subgroup framework, examining baseline systolic blood pressure as a clinically relevant modifier of treatment effect. Specifically, this analysis integrates clinical outcomes, quality-of-life measures, and longitudinal medication utilization to address a real-world therapeutic dilemma: management of angina in patients with low blood pressure who have limited tolerance to guideline-recommended anti-anginal therapies. To our knowledge, this is the first ISCHEMIA secondary analysis to systematically evaluate the interaction between baseline SBP, revascularization strategy, medication patterns, and long-term outcomes.

The management of CCS primarily aims to achieve two objectives: alleviating angina symptoms and enhancing patients’ quality of life; and secondly, lowering the risk of major cardiovascular events [15,23]. From this perspective, our study provides evidence supporting the effectiveness of invasive treatment strategies in patients with low blood pressure. In the secondary endpoint, the higher rate of heart failure hospitalization observed in the invasive strategy group should be interpreted cautiously and does not necessarily indicate a true increase in heart failure incidence. Several alternative explanations merit consideration. Firstly, patients assigned to an invasive strategy underwent more intensive cardiovascular evaluation and follow-up, which may have led to a lower threshold for diagnosing and hospitalizing heart failure events (surveillance or ascertainment bias). Secondly, early discontinuation or down-titration of anti-anginal or antihypertensive medications after revascularization may have altered volume status or neurohormonal balance in susceptible individuals, increasing the likelihood of heart failure-related hospitalization without reflecting progressive myocardial dysfunction. Finally, given the post hoc nature of this analysis and the relatively small number of heart failure events, this finding may represent a chance imbalance rather than a causal effect.

Selecting 120 mmHg as the cut-off value for distinguishing high and low SBP is reasonable, because of the J curve, an excessive reduction in blood pressure may increase the incidence of adverse outcomes, particularly among patients at high cardiovascular risk, where existing organ damage may compromise autoregulatory mechanisms responsible for maintaining adequate vital organ perfusion during blood pressure decline [11,24]. Despite prior reports indicating that an elevated risk of myocardial infarction associated with reductions in systolic BP below 120–130 mm Hg has been consistently documented among patients with a history of cardiac disease [25], in our study, the incidence of MI as a secondary endpoint was not higher in the low SBP group compared with the high SBP group (Table 3, Table 5 and Table 6, 5.68% vs. 8.40%). The apparent reduction in myocardial infarction observed in the low systolic blood pressure subgroup should be viewed as a subgroup-specific causal estimate with limited precision, rather than as definitive evidence of benefit. Notably, a marked reduction in the rate of primary endpoints was observed in the invasive treatment subgroup (Figure 3), which may be attributed to the overall benefits of the revascularization strategy [26,27]. Park et al. demonstrated a J-shaped relationship between baseline blood pressure and long-term outcomes in patients undergoing PCI, with nadir values of 119 mmHg for SBP and 74 mmHg for DBP, findings that are consistent with our study [28]. The observation highlighting the need to avoid excessively low blood pressure levels, which may be associated with adverse in-hospital and long-term events, further limited the use of anti-anginal drugs with vasodilatory properties [29]. In our study, the myocardial infarction rate in the PCI strategy was lower within the low SBP subgroup, which may partially account for the observed subgroup-specific differences, but the association between low SBP and PCI represents a subgroup-specific causal contrast based on randomized treatment assignment and should be interpreted cautiously given the exploratory nature of the analysis.

In clinical practice, side effects are the leading cause of treatment discontinuation or poor adherence, which is well known to diminish or even negate the therapeutic benefits of the intervention, but maintaining high medication adherence leads to more favorable clinical results [30,31]. Therefore, in patients with angina and concomitant hypotension, it is reasonable to employ anti-anginal agents that have minimal impact on blood pressure or lack antihypertensive effects altogether, in order to avoid further reductions in blood pressure [20,32], which was consistent with the results of our study that the low SBP (≤120) group had the low proportion of patients taking ACEI/ARB, β-blockers, and CCBs, and a similar proportion taking long-acting nitrates (Table 7). Therefore, in patients with CCS and low SBP (especially for patients with a history of myocardial infarction, a total of 17.8% in our research), the addition of first-line anti-anginal agents such as β-blockers and CCBs or prognostic benefit drugs like β-blockers and ACEI/ARBs is challenging [33,34]. Theoretically, the management of such patients may rely on revascularization therapy, and our study supports this concept, demonstrating that patients in the low SBP group achieved greater clinical benefits over 3 years and reported a higher quality of life. The potential biological plausibility linking lower systolic blood pressure, limited pharmacologic tolerance, and a greater apparent benefit from revascularization warrants further consideration. Patients with lower baseline systolic blood pressure may have reduced hemodynamic reserve and are often less able to tolerate vasodilatory or negatively inotropic anti-anginal therapies, such as nitrates, beta-blockers, or calcium channel blockers, due to symptomatic hypotension. In this context, symptom control with optimal medical therapy may be constrained, and relief of myocardial ischemia through revascularization may offer an alternative therapeutic pathway that does not rely on further blood pressure reduction. Accordingly, in patients with low systolic blood pressure, an invasive strategy may theoretically provide symptomatic and ischemic benefit by improving myocardial perfusion rather than intensifying pharmacologic vasodilation. Nevertheless, this proposed mechanism remains speculative and should be interpreted within the post hoc and exploratory nature of the present subgroup analysis.

Although a small baseline imbalance in SAQ-7 Quality of Life scores was observed, treatment assignment was randomized, and the magnitude of the difference was modest. Baseline health-related quality of life may nonetheless influence symptom perception and response to revascularization, and thus could act as a potential effect modifier. The INV group experienced better quality of life at the 3-year follow-up, with a higher SAQ score compared with the CON group, which was consistent with a previous study. Post hoc analyses of the ISCHEMIA trial demonstrated that complete revascularization was associated with a significant improvement in the SAQ Angina Frequency score, particularly among patients experiencing more frequent angina episodes, compared with the CON group and the incomplete revascularization group [35]. The findings of the present analysis should also be interpreted in the context of the ORBITA and ORBITA-2 trials, which specifically evaluated the symptomatic benefits of percutaneous coronary intervention (PCI) in patients with stable angina. The original ORBITA trial was a double-blind, randomized, placebo-controlled study comparing PCI with a sham procedure in patients with stable coronary artery disease receiving optimized medical therapy, and it demonstrated no significant improvement in exercise time or angina-related symptoms with PCI over placebo. In contrast, the ORBITA-2 trial was designed to evaluate the effect of PCI on angina relief in patients with more severe and frequent anginal symptoms, conducted in the absence of background anti-anginal medications, thereby isolating the symptomatic effect of revascularization. In ORBITA-2, PCI was associated with a significant reduction in angina frequency compared with placebo, highlighting the importance of symptom burden and pharmacologic context in determining the clinical benefit of PCI. Notably, both ORBITA and ORBITA-2 primarily focused on short-term symptom relief rather than long-term clinical outcomes, and neither study was designed to assess treatment effect modification by systolic blood pressure. In contrast, the present post hoc analysis of the ISCHEMIA trial explores the interaction between baseline systolic blood pressure and treatment strategy with respect to clinical cardiovascular outcomes. Taken together, these findings suggest that the potential benefits of an invasive strategy may depend on patient-specific clinical characteristics, including symptom burden, hemodynamic profile, and tolerance to anti-anginal therapy, and underscore the need for prospective studies integrating these factors [36,37]. The sub-analysis of ORBITA-2 further confirmed this finding, showing that the more typical the angina symptoms, the greater the symptomatic relief achieved with PCI (e.g., in cases of Rose angina). Moreover, the study found no clear association between symptom severity and either the underlying nature of the disease or its anatomical severity [4].

In addition to hemodynamic parameters such as systolic blood pressure, myocardial viability assessment plays an important role in clinical decision-making for patients with chronic coronary syndromes. Viability imaging helps distinguish dysfunctional but viable myocardium from irreversible scar tissue, thereby informing the potential benefit of revascularization [38]. Cardiac magnetic resonance imaging (MRI), particularly with late gadolinium enhancement, provides accurate characterization of scar burden and viable myocardium and offers incremental prognostic value. Other modalities, including stress echocardiography, SPECT, and PET, may also contribute depending on clinical context. Although myocardial viability was not evaluated in the present analysis, integrating viability assessment with hemodynamic status and symptom burden may further improve individualized risk stratification and selection of patients most likely to benefit from an invasive strategy. Future studies incorporating systematic viability assessment alongside blood pressure stratification are warranted [39].

### Limitations

Our study demonstrated the greatest benefit only in the invasive treatment group when stratified by SBP, without incorporating DBP into the analysis. However, as a primary determinant of coronary perfusion pressure, the role of DBP in long-term prognosis cannot be overlooked. For example, an Australian registry study (Hypertension) showed that among patients with well-controlled SBP, lower DBP levels were associated with a higher incidence of long-term adverse events, with DBP < 50 mmHg identified as an independent predictor of all-cause mortality (HR = 1.55, 95%CI 1.20 to 2.00) [40]. This finding suggests that excessively low DBP may compromise coronary and microvascular perfusion pressure. The concept of intolerance to anti-anginal medications was inferred rather than formally assessed, which reflects the clinical tendency toward lower use or dose limitation of vasodilatory anti-anginal therapies in patients with lower systolic blood pressure. Our study did not further investigate or assess the specific blood pressure levels within the low SBP group (e.g., the proportion of patients with SBP < 100 mmHg), nor did it evaluate the impact of dynamic blood pressure fluctuations on prognosis [41]. The limited sample size of this study may have hindered the detection of subtle differences in the primary endpoint between groups. Although the Cox regression model accounted for multiple covariates, the possibility of residual confounding cannot be ruled out. Finally, although treatment assignment was randomized, subgroup analyses may still be subject to residual confounding due to imbalances within subgroups. The exploratory and post hoc nature of the subgroup analyses raises the possibility of chance findings, particularly in the context of multiple comparisons, and the statistical power for interaction testing was limited. Post hoc subgroup analyses are inherently susceptible to type I error. Accordingly, these findings should be interpreted as hypothesis-generating and should not directly guide clinical practice without confirmation in prospective, adequately powered randomized studies.

## 5. Conclusions

In patients with stable angina pectoris, the invasive treatment group demonstrated better quality of life at the 3-year follow-up. Among low SBP (≤120 mmHg) patients who were unable to tolerate anti-anginal drugs with vasodilatory properties, the invasive strategy group showed a lower incidence of adverse events over the 3-year period, especially hospitalization for myocardial infarction, highlighting the potential benefits of individualized treatment strategies for these patients. However, this subgroup-specific causal contrast derives from a post hoc exploratory analysis and should be interpreted cautiously; prospective randomized studies are warranted to confirm these findings.

## Figures and Tables

**Table 1:** Baseline characteristics of the participants with angina at baseline by treatment strategies.

	All Participants (N = 3544)	Conservative Treatment (N = 1743)	Invasive Treatment (N = 1801)	*p*-Value
Demographics				
Age, years	62.62 (9.54)	62.63 (9.67)	62.61 (9.43)	0.958
Male sex	75.3 (2669/3544)	76.2 (1328/1743)	74.5 (1341/1801)	0.248
Race or ethnic group				
Hispanic or Latino	13.1 (463/3544)	13.4 (234/1743)	12.7 (229/1801)	0.550
Not Hispanic or Latino	79.7 (2824/3544)	79.7 (1390/1743)	79.6 (1434/1801)	
Unknown	7.3 (257/3544)	6.8 (119/1743)	7.7 (138/1801)	
Cigarette smoking				
Current smoker	14.4 (510/3544)	243/1743 (13.9)	14.8 (267/1801)	0.148
Former smoker	40.8 (1447/3544)	42.1 (734/1743)	39.6 (713/1801)	
Never smoked	44.6 (1581/3544)	43.7 (761/1743)	45.5 (820/1801)	
Unknown	0.2 (6/3544)	0.3 (5/1743)	0.1 (1/1801)	
Clinical history				
Hypertension	71.4 (2519/3530)	71.2 (1237/1737)	71.5 (1282/1793)	0.878
Diabetes	41.3 (1464/3544)	41.3 (720/1743)	41.3 (744/1801)	1.000
Prior myocardial infarction	17.8 (629/3533)	17.8 (309/1736)	17.8 (320/1797)	0.631
Prior PCI	18.2 (644/3541)	17.7 (309/1741)	18.6 (335/1800)	0.667
Prior CABG	130 (3.7)	63 (3.6)	67 (3.7)	0.938
Heart failure	3.7 (131/3544)	3.4 (59/1743)	4.0 (72/1801)	0.380
Peripheral vascular disease	3.3 (116/3537)	2.9 (50/1738)	3.7 (66/1799)	0.208
Cerebrovascular disease	6.6 (233/3535)	6.3 (110/1738)	6.8 (123/1797)	0.768
Vital signs and laboratory values				
Systolic blood pressure, mmHg	132.26 (17.62)	131.72 (17.32)	132.79 (17.89)	0.071
Diastolic blood pressure, mmHg	76.39 (10.38)	76.22 (10.34)	76.56 (10.42)	0.331
Heart rate, bpm	68.41 (11.01)	68.07 (11.08)	68.74 (10.93)	0.073
Body mass index, kg/m^2^	27.78 (5.23)	27.94 (5.43)	27.62 (5.03)	0.070
LDL-C, mg/dL	92.37 (39.52)	92.52 (38.81)	92.22 (40.21)	0.825
Non-HDL-C, mg/dL	118.40 (44.54)	118.58 (44.02)	118.23 (45.04)	0.819
Stress test and cardiac imaging findings				
Left ventricular ejection fraction, %	60.37 (8.03)	60.18 (8.13)	60.56 (7.92)	0.163
Left ventricular systolic dysfunction (left ventricular ejection fraction ≥35% or <45%)	4.0 (143/3540)	4.1 (72/1741)	3.9 (71/1799)	0.960
Degree of ischemia on stress testing, as assessed by core laboratory				
None	4.5 (160/3532)	4.8 (84/1738)	4.2 (76/1797)	0.504
Mild	6.6 (232/3532)	6.2 (107/1738)	7.0 (125/1797)	
Moderate	29.8 (1051/3532)	28.9 (502/1738)	30.6 (549/1797)	
Severe	57.8 (2040/3532)	58.5 (1017/1738)	56.9 (1023/1797)	
Uninterpretable	1.4 (49/3532)	1.4 (25/1738)	1.3 (24/1797)	
Modality of stress testing				
Nuclear SPECT	44.1 (1563/3544)	45.1 (786/1743)	43.1 (777/1801)	0.665
Echocardiography	19.9 (707/3544)	19.4 (338/1743)	20.5 (369/1801)	
Cardiac magnetic resonance	4.8 (169/3544)	4.8 (84/1743)	4.7 (85/1801)	
Exercise tolerance test	31.2 (1105/3544)	30.7 (535/1743)	31.6 (570/1801)	
CCTA findings				
No. of diseased vessels (≥50% stenosis)				
0	0.1 (3/2059)	0.0 (1/1015)	0.2 (2/1044)	0.243
1	23.9 (493/2059)	23.5 (239/1015)	24.3 (254/1044)	
2	30.7 (632/2059)	33.2 (337/1015)	28.3 (295/1044)	
≥3	45.2 (931/2059)	43.2 (438/1015)	47.2 (493/1044)	
Proximal LAD ≥ 50% stenosis	46.2 (1226/2653)	47.3 (620/1311)	45.2 (606/1342)	0.659
Angina characteristic				
SAQ-7 Physical Limitation Score	73.65 (24.12)	74.25 (24.35)	73.08 (23.88)	0.196
SAQ Angina Frequency score	71.33 (17.49)	71.86 (17.13)	70.81 (17.83)	0.101
SAQ-7 Quality of Life Score	50.00 (38.00, 75.00)	50.00 (38.00, 75.00)	50.00 (38.00, 63.00)	0.045
SAQ-7 Summary Score	65.63 (16.90)	66.22 (16.91)	65.05 (16.87)	0.057
Daily or weekly angina	31.4 (934/2977)	29.9 (440/1472)	32.8 (494/1505)	0.319

Note: Data are presented as mean (standard deviation), median (p25, p75), or % (number/total number). All analyses shown represent post hoc secondary analyses of the ISCHEMIA randomized clinical trial.

**Table 2:** Estimated difference between treatment groups in cumulative event rates.

Variable	Invasive Strategy (N = 1801)	Conservative Strategy (N = 1743)	Estimated Difference (95%CI)	Hazard Ratio (95%CI)
No. of patients with events	192	201	-	-
Cumulative event rate (%)				0.94 (0.77 to 1.14)
At 6 mo	5.7	4.0	1.7 (0.2, 3.1)	
At 1 yr	7.4	5.7	1.7 (0.1, 3.4)	
At 2 yr	9.3	9.4	−0.1 (−2.1, 1.8)	
At 3 yr	10.7	11.5	−0.8 (−3.0, 1.2)	

All analyses shown represent post hoc secondary analyses of the ISCHEMIA randomized clinical trial.

**Table 3:** Secondary endpoint at 3 yr follow-up (cumulative event rates for each point).

Variable	Invasive Strategy (N = 1801)	Conservative Strategy (N = 1743)	Unadjusted HR (95%CI)	*p*-Value	Adjusted HR (95%CI)	*p*-Value
CV death	45 (2.50)	52 (2.98)	0.84 (0.57–1.26)	0.3996	0.87 (0.59–1.30)	0.5041
Unstable angina	14 (0.78)	23 (1.32)	0.59 (0.30–1.15)	0.1210	0.58 (0.30–1.12)	0.1028
Heart failure	21 (1.17)	8 (0.46)	2.56 (1.14–5.79)	0.0235	2.58 (1.19–5.73)	0.0204
Resuscitation after cardiac arrest	2 (0.11)	4 (0.23)	0.49 (0.09–2.65)	0.4036	0.53 (0.10–2.87)	0.4625
MI	135 (7.50)	134 (7.69)	0.99 (0.78–1.26)	0.9512	0.998 (0.79–1.27)	0.9900

Note: The Cox model was adjusted for age, sex, degree of ischemia, smoking status, hypertension, diabetes, prior MI, PCI, or CABG, history of HF, and continuous ejection fraction. CV, cardiovascular death; MI, myocardial infarction. All analyses shown represent post hoc secondary analyses of the ISCHEMIA randomized clinical trial.

**Table 4:** Angina characteristic at the 3-year follow-up.

Angina Characteristic-Follow Up	All Participants	Conservative Treatment	Invasive Treatment	*p*-Value
SAQ-7 Physical Limitation Score	85.97 (20.97)	84.26 (22.24)	87.66 (19.48)	0.0023
SAQ Angina Frequency score	91.63 (14.21)	90.73 (14.50)	92.50 (13.88)	0.0131
SAQ-7 Quality of Life Score	85.05 (16.01)	83.56 (16.49)	86.50 (15.40)	0.0002
SAQ-7 Summary Score	77.78 (22.96)	75.70 (23.46)	79.82 (22.29)	0.0003
Daily or weekly angina	6.82 (109/1599)	7.22 (57/790)	6.43 (52/809)	0.5322

SAQ, Seattle Angina Questionnaire. All analyses shown represent post hoc secondary analyses of the ISCHEMIA randomized clinical trial.

**Table 5:** Vital signs analyses for the primary endpoint (point estimates and confidence intervals are based on covariate adjusted Cox regression analyses).

	Percent of Patients	Estimated 3 Yr Event Rate						Difference in Event Rate, Invasive Strategy Minus Conservative Strategy (95% CI)	HR (95%CI)	*p* for Interaction
		Invasive Strategy			Conservative Strategy					
		N	events	%	N	events	%			
Heart Rate, bpm										
≤60	27.20	469	44	9.38	495	44	8.89	0.49 (−3.15–4.13)	1.14 (0.75–1.74)	0.3775
>60	72.80	1332	148	11.11	1248	157	12.58	−1.47 (−3.97–1.03)	0.90 (0.72–1.13)	
SBP, mmHg										
≤120	29.80	527	35	6.64	529	59	11.15	−4.51 (−7.93–−1.09)	0.58 (0.38, 0.89)	0.0162
>120	70.20	1274	157	12.32	1214	142	11.70	0.63 (−1.93–3.18)	1.08 (0.86–1.36)	
DBP, mmHg										
≤80	74.86	1333	140	10.50	1320	151	11.44	−0.94 (−3.32–1.44)	0.93 (0.74–1.18)	0.8545
>80	25.14	468	52	11.11	423	50	11.82	−0.71 (−4.90–3.48)	0.98 (0.67–1.46)	

Note: The Cox model was adjusted for age, sex, degree of ischemia, smoking status, hypertension, diabetes, prior MI, PCI, or CABG, history of HF, and continuous ejection fraction; All analyses shown represent post hoc secondary analyses of the ISCHEMIA randomized clinical trial. Subgroup interaction analyses based on heart rate, systolic blood pressure, and diastolic blood pressure were exploratory and post hoc in nature, and no formal adjustment for multiple comparisons was performed.

**Table 6:** The component of primary endpoints for 3-year follow-up of patients with low SBP at baseline.

Variable	Invasive Strategy (N = 527)	Conservative Strategy (N = 529)	*p*-Value	Unadjusted HR (95%CI)	*p*-Value	Adjusted HR (95%CI)
CV death	9 (1.71)	17 (3.21)	0.1152	0.52 (0.23–1.17)	0.1134	0.51 (0.22–1.18)
Unstable angina	3 (0.57)	6 (1.13)	0.3290	0.50 (0.13–2.01)	0.3848	0.53 (0.13–2.23)
Heart failure	3 (0.57)	1 (0.19)	0.3389	3.02 (0.31–29.06)	0.2462	3.25 (0.44–23.76)
Resuscitation after cardiac arrest	0 (0)	3 (0.57)	<0.0001	-	<0.0001	-
MI	21 (3.98)	39 (7.37)	0.0293	0.59 (0.37–0.95)	0.0255	0.58 (0.36–0.94)

Note: The Cox model was adjusted for age, sex, degree of ischemia, smoking status, hypertension, diabetes, prior MI, PCI, or CABG, history of HF, and continuous ejection fraction. All analyses shown represent post hoc secondary analyses of the ISCHEMIA randomized clinical trial.

**Table 7:** Baseline and last visit medical therapy attainment according to baseline systolic blood pressure.

	SBP ≤ 120 mmHg (N = 1056)	SBP 120–140 mmHg (N = 1603)	SBP > 140 mmHg (N = 885)	*p*-Value
Baseline				
Age, years	60.6 (9.9)	62.61 (9.32)	65.05 (9.00)	<0.001
Male, %	817 (77.4)	1212 (75.6)	640 (72.3)	0.034
Taking aspirin or aspirin alternative	957 (90.6)	1452 (90.6)	789 (89.2)	0.022
Taking high-intensity statin	417 (39.5)	634 (39.6)	317 (35.8)	0.037
Medically indicated to take ACE/ARB	769 (72.8)	1332 (83.1)	787 (88.9)	<0.001
Met individual ACE/ARB goal	499 (47.3)	984 (61.4)	562 (63.5)	<0.001
Patients who need to take ACEI/ARB and follow the required dosage ratio (quotient of the two variables above)	0.649	0.739	0.714	<0.001
Taking ACEI/ARB	596 (56.4)	1072 (66.9)	602 (68.0)	<0.001
Medically indicated to take β-blocker	202 (19.1)	313 (19.5)	133 (15.0)	0.002
Met individual β-blocker	181 (17.1)	280 (17.5)	119 (13.4)	0.077
Patients who need to take β-blocker and follow the required dosage ratio (quotient of the two variables above)	0.896	0.895	0.895	0.998
Taking β-blocker	888 (84.1)	1327 (82.8)	697 (78.8)	<0.001
Taking CCB	244 (23.1)	511 (31.9)	341 (38.5)	<0.001
Taking anti-anginal medications	532 (50.4)	721 (45.0)	392 (44.3)	0.001
Taking long-acting nitrates	451 (42.7)	596 (37.2)	334 (37.7)	0.001
Adherent to medications based on Morisky–Green–Levine assessment	734 (69.5)	1112 (69.4)	633 (71.5)	0.653
Last visit				
Taking aspirin or aspirin alternative	546 (51.7)	840 (52.4)	454 (51.3)	0.901
Taking high-intensity statin	383 (36.3)	578 (36.1)	317 (35.8)	0.888
Medically indicated to take ACE/ARB	436 (41.3)	793 (49.5)	459 (51.9)	<0.001
Met individual ACE/ARB goal	273 (25.9)	575 (35.9)	348 (39.3)	<0.001
Patients who need to take ACEI/ARB and follow the required dosage ratio (quotient of the two variables above)	0.626	0.725	0.758	<0.001
Taking ACEI/ARB	344 (32.6)	643 (40.1)	378 (42.7)	<0.001
Medically indicated to take β-blocker	132 (12.5)	202 (12.6)	87 (9.8)	0.238
Met individual β-blocker	108 (10.2)	159 (9.9)	74 (8.4)	0.319
Patients who need to take β-blocker and follow the required dosage ratio (quotient of the two variables above)	0.818	0.787	0.851	0.433
Taking β-blocker	476 (45.1)	721 (45.0)	405 (45.8)	0.767
Taking CCB	119 (11.3)	327 (20.4)	241 (27.2)	<0.001
Taking anti-anginal medications	196 (18.6)	301 (18.8)	162 (18.3)	0.971
Taking long-acting nitrates	150 (14.2)	237 (14.8)	127 (14.4)	0.975
Adherent to medications based on Morisky–Green–Levine assessment	398 (37.7)	621 (38.7)	327 (36.9)	0.300

Note: All categorical data are presented as number (%). All analyses shown represent post hoc secondary analyses of the ISCHEMIA randomized clinical trial. SBP, systolic blood pressure

**Table 8:** Baseline and last visit medical therapy outcome in patients with SBP ≤ 120 mmHg.

	Invasive Strategy (N = 527)	Conservative Strategy (N = 529)	*p*-Value
Baseline			
Age, years	60.24 (9.96)	60.94 (9.76)	0.245
Male, %	399 (75.7)	418 (79.0)	0.226
Taking aspirin or aspirin alternative	472 (89.6)	485 (91.7)	0.485
Taking high-intensity statin	203 (38.5)	214 (40.5)	0.813
Medically indicated to take ACE/ARB	382 (72.5)	387 (73.2)	0.5
Met individual ACE/ARB goal	254 (48.2)	245 (46.3)	0.669
Patients who need to take ACEI/ARB and follow the required dosage ratio (quotient of the two variables above)	0.66	0.63	0.355
Taking ACEI/ARB	301 (57.1)	295 (55.8)	0.906
Medically indicated to take β-blocker	99 (18.8)	103 (19.5)	0.493
Met individual β-blocker	89 (16.9)	92 (17.4)	0.952
Patients who need to take β-blocker and follow the required dosage ratio (quotient of the two variables above)	0.90	0.89	0.893
Taking β-blocker	440 (83.5)	448 (84.7)	0.866
Taking CCB	121 (23.0)	123 (23.3)	0.994
Taking anti-anginal medications	245 (46.5)	287 (54.3)	0.041
Taking long-acting nitrates	208 (39.5)	243 (45.9)	0.104
Adherent to medications based on Morisky–Green–Levine assessment	365 (69.3)	369 (69.8)	0.378
Last visit			
Taking aspirin or aspirin alternative *	269 (51.0)	277 (52.4)	0.787
Taking high-intensity statin ^#^	190 (36.1)	193 (36.5)	0.963
Medically indicated to take ACE/ARB	214 (40.6)	222 (42.0)	0.305
Met individual ACE/ARB goal	132 (25.0)	141 (26.7)	0.819
Patients who need to take ACEI/ARB and follow the required dosage ratio (quotient of the two variables above)	0.62	0.64	0.693
Taking ACEI/ARB	171 (32.4)	173 (32.7)	0.993
Medically indicated to take β-blocker ^&^	65 (12.3)	67 (12.7)	0.673
Met individual β-blocker	52 (9.9)	56 (10.6)	0.354
Patients who need to take β-blocker and follow the required dosage ratio (quotient of the two variables above)	0.80	0.84	0.594
Taking β-blocker	232 (44.0)	244 (46.1)	0.455
Taking CCB	56 (10.6)	63 (11.9)	0.783
Taking anti-anginal medications	65 (12.3)	131 (24.8)	<0.001
Taking long-acting nitrates	50 (9.5)	100 (18.9)	<0.001
Adherent to medications based on Morisky–Green–Levine assessment	201 (38.1)	197 (37.2)	0.662

Note: All categorical data are presented as number (%). All analyses shown represent post hoc secondary analyses of the ISCHEMIA randomized clinical trial. * This variable indicates whether or not the participant met the OMT goal of being on aspirin or an aspirin alternative medication at the corresponding visit indicated by the VISITID variable. A participant is defined as meeting the OMT goal of being on aspirin or an aspirin alternative medication if they are indicated as taking an anti-platelet medication. Otherwise, if they are indicated as not taking an anti-platelet medication and not taking an anticoagulant medication, then they are defined as not missing this goal. Otherwise, the participant is missing information on whether or not this goal is met. ^#^ This variable indicates whether or not the participant met the OMT goal of being on a high-intensity dose of either rosuvastatin or atorvastatin at the corresponding visit indicated by the VISITID variable. If a participant is indicated as not taking a high-intensity dose of either rosuvastatin or atorvastatin or the participant is indicated as not taking rosuvastatin or atorvastatin, then they are defined as not being on a high-intensity dose of either rosuvastatin or atorvastatin. Otherwise, the participant is missing information on whether or not this goal is met. ^&^ This variable indicates whether or not a participant has met the OMT goal of being on a beta-blocker medication at the corresponding visit indicated by the VISITID variable regardless of whether or not they are indicated to be taking a beta-blocker medication.

## Data Availability

The data that support the findings of this study are available from the NHLBI Biologic Specimen and Data Repository Information Coordinating Center but restrictions apply to the availability of these data, which were used under license for the current study, and so are not publicly available.

## Supplementary Materials

The following supporting information can be downloaded at: https://www.mdpi.com/article/10.3390/jcm15062100/s1, Central illustration. Reference [42] is cited in the Supplementary Materials.
